# Enzymic Activity, Metabolites, and Hematological Responses Changes of Clinical Healthy High-Risk Beef Calves During Their First 56-Days from Arrival

**DOI:** 10.3390/ani15020133

**Published:** 2025-01-08

**Authors:** Octavio Carrillo-Muro, Pedro Hernández-Briano, Paola Isaira Correa-Aguado, Alejandro Rivera-Villegas, Oliver Yaotzin Sánchez-Barbosa, Rosalba Lazalde-Cruz, Alberto Barreras, Alejandro Plascencia, Daniel Rodríguez-Cordero

**Affiliations:** 1 Unidad Académica de Medicina Veterinaria y Zootecnia, Universidad Autónoma de Zacatecas, General Enrique Estrada 98500, Mexico; octavio_cm@uaz.edu.mx (O.C.-M.); pedro.hernandez@uaz.edu.mx (P.H.-B.); paocorrea@uaz.edu.mx (P.I.C.-A.); alejandro.rivera@uaz.edu.mx (A.R.-V.); oliver.sanchez@uaz.edu.mx (O.Y.S.-B.); 2 Instituto de Investigaciones en Ciencias Veterinarias, Universidad Autónoma de Baja California, Mexicali 21100, Mexico; rosalba.lazalde@uabc.edu.mx (R.L.-C.); beto_barreras@yahoo.com (A.B.); 3 Facultad de Medicina Veterinaria y Zootecnia, Universidad Autónoma de Sinaloa, Culiacán 80260, Mexico

**Keywords:** blood cells, calcium, electrolytes, glucose, platelets, newly received, lipids, protein

## Abstract

The objective of this study was to evaluate the changes in enzymic activity, metabolites, and hematological responses during the first 56-d of arrival of newly received calves, which were qualified at reception as high-risk but diagnosed as clinically healthy. A total of 320 blood samples were taken from 64 crossbred bull calves (average initial body weight = 148.3 ± 1.3 kg) at different times from arrival (d 0, 14, 28, 42, and 56 of received). Calves included in the study were received in June (*n* = 20), November (*n* = 24), and April (*n* = 20); thus, experimental treatments were arranged in a generalized complete block design (three blocks = month of arrival). The following parameters were determined: total white blood cells (WBC): lymphocytes (LYM), lymphocytes % (LYM%), monocytes (MON), monocytes % (MON%), granulocytes (GRA), granulocytes % (GRA%), platelets (PLT), and mean platelet volume (MPV); red blood cells (RBC): red blood cell distribution width test % (RDW%), hematocrit (HCT), and mean corpuscular volume (MCV); hemoglobin (HGB): mean corpuscular hemoglobin (MCH) and mean corpuscular hemoglobin concentration (MCHC). The enzymatic activity and metabolites analyzed were alkaline phosphatase (ALP), gamma glutamyltransferase (GGT), aspartate aminotransferase (AST), alanine aminotransferase (ALT), total protein (TP), albumin (ALB), globulin (GLO), ALB/GLO ratio, blood urea nitrogen (BUN), creatinine (CRE), total bilirubin (TBIL), total cholesterol (TCHO), triglycerides (TG); (4) calcium (Ca), glucose (GLU), sodium (Na^+^), potassium (K^+^), and chlorine (Cl^−^). It was observed that ALP, ALT, TP, ALB, GLO, ALB/GLO ratio, TCHO, TG, Ca, and GLU increased as days from reception increased (linear effect, *p* ≤ 0.04), whereas CRE and TBIL were reduced (linear effect, *p* ≤ 0.02). A quadratic response (*p* ≤ 0.001) was observed to GGT and AST values being maximal on days 1 and 56 after arrival (*p* ≤ 0.001). Na^+^, K^+^, and Cl^−^ concentrations were not affected by prolonged days after arrival. Finally, blood cells of LYM, LYM%, PLT, RBC, HGB, HCT%, MCV, and MCH increased (linear effect, *p* ≤ 0.001) as the number of days after arrival increased. Whereas MON% was linearly decreased (*p* ≤ 0.05). It was concluded that even when all parameters were within the range of reference intervals (RIs) determined for healthy cattle, during the period of monitoring, as the days after arrival lengthened, blood serum parameters related to health and immunity increased, and metabolites related to tissue injury decreased. In contrast, plasmatic electrolytes (Na^+^, K^+^, and Cl^−^) were slightly reduced as the day after arrival increased. Apparently, at least 42 d is the minimum period after arrival to permit calves to reach more adequate physiological and metabolic conditions before starting the fattening phase.

## Simple Summary

Quantification of enzymic activity, metabolites, and hematological responses is useful for determining the physiological, nutritional, metabolic, and clinical status of high-risk beef calves. In the initial days after calves arrive at the feedlot, they regain lost water and body weight, stabilize or improve their immunity, and establish a social structure. The ruminal microorganisms are adapted to grain-based diets; therefore, it is expected that blood and serum parameters change during the initial days after arrival. However, to date, information on changes in health indicators, such as blood and serum parameters, during adaptation in the first 56 d of arrival is scarce. According to the results of this study, the more days at reception, the more blood and serum parameter values related to health and immunity were improved, whereas the concentration of blood parameters related to tissue injury was minimized; this behavior indicates an improvement in the physiological, nutritional, metabolic, and clinical status of high-risk beef calves during their stay. Apparently, at least 42 d is the minimum period after arrival to permit calves to reach more adequate physiological and metabolic conditions before starting the fattening phase.

## 1. Introduction

The main objectives of the receiving phase in feedlots are to preserve lower morbidity and mortality and to recover the purchase weight in the shortest time. This is because 28% of cattle placed in North American feedlots are considered high-risk [1], which makes the reception of beef calves a very important challenge since it directly impacts the economy and animal welfare in the beef cattle industry. At this stage (first 42 or 56 d), the morbidity rate can reach up to 16%, and the mortality rate is highest compared to the transition (phase following the reception, in which the target is that the cattle be gradually adapted to high-grain rations), and the fattening phase, in which cattle are fed with high-grain diets up to reach the desired slaughter weight [2,3].

Regarding the reception phase, in practice, an increase in dry matter consumption and weight recovery are the main indicators of the adaptation of clinically healthy newly arrived cattle to the fattening process. However, the relationship between enzymatic activity, metabolites, and hematological responses and the health of beef cattle has been increasingly elucidated. In this sense, several active molecules and metabolites have been identified as “markers” of homeostasis, immunity, and nutritional status that reflect the health and productivity of beef cattle [4,5]. Hence, the quantification of enzymic activity, metabolites, and hematological responses is more precise and useful for determining the physiological, nutritional, metabolic, and clinical status of high-risk beef calves. A few days after calves arrive at the feedlot, clinically healthy calves regain lost water and body weight, stabilize or improve their immunity, and establish a social structure. In addition, ruminal microorganisms are adapted to grain-based diets, favoring that the nutritional requirements are met, therefore, it is expected that blood and serum parameters change over time. However, to date, information on changes in health indicators, such as blood and serum parameters, during the adaptation of clinically healthy calves in the first 56 d of arrival is scarce.

The majority of studies have been performed to evaluate enzymatic and physiological responses as a result of implanting administration time upon arrival [6], supplementation with probiotics or glucogenic feed additives [7], times at vaccine application [8], or administration of different types of metaphylaxis [9]. Surprisingly, there are no reports regarding the natural evolution (without treatment) of blood enzymatic and physiological parameters of calves that qualify as clinically healthy upon arrival at the feedlot. This information will more precisely determine the adequate time for the physiological and metabolic conditions of newly arrived calves before starting the transition phase and prior to final fattening. Therefore, the objective of this study was to determine the effect of days from arrival (0, 14, 28, 42, and 56) on enzymatic activity, metabolites, and hematological responses in a clinically healthy population of high-risk beef calves.

## 2. Materials and Methods

This work was approved by the UAMVZ-UAZ—Institutional Bioethics and Animal Welfare Committee (Protocol # 16 May 2023) and adhered to the Official Mexican Standards: (1) NOM-051-ZOO-1995, Humanitarian care in the mobilization of animals; (2) NOM-062-ZOO-1999, Humane care and welfare of experimental animals; and (3) NOM-024-ZOO-1995, Animal health stipulations and characteristics during animal transportation. The experiment was conducted from June 2023 to April 2024 at the Torunos Livestock Preconditioning Center, within the experimental area, owned by Grupo Exportador Pa Lante S.P.R. de R.L., located 18 km east of Fresnillo, Zacatecas, Mexico. Arrival inclusion criteria, processing, feeding, clinical, and health management data collection procedures were exactly the same as those described previously by Carrillo-Muro et al. [10]. Briefly, arrival processing included vaccination against clostridial and *Mannheimia haemolytica* pathogens (Biovac 11 Vías^®^, Biozoo, Jalisco, Mexico), treatment for internal parasites (4% ivermectin, Master LP^®^ injectable, Ourofino Salud Animal, São Paulo, Brazil) and external parasites (pour on cypermethrin, Cypermil Pour On^®^, Ourofino Salud Animal, São Paulo, Brazil) and antimicrobial metaphylaxis with oxytetracycline (Emicina^®^, Zoetis, Ciudad de Mexico, Mexico). The diet (type reception diet) was the same for all cattle. The total mixed diet offered contained 14.5% crude protein (CP) and 34.8% neutral detergent fiber, providing 0.98 Mcal net energy for gain/kg. The diet included 50% roughage (mature alfalfa hay and oats hay in equal proportion), along with 28% cracked corn grain, 10.5% soybean meal, 5% cane molasses, 2.5% soybean oil, and the remaining 4% was composed of mineral supplements and vitamin supplements. The number of days on feed was 56. Cattle were evaluated daily for health and well-being by an experienced veterinarian, and medical treatment followed a preplanned case definition.

### 2.1. Experimental Design and Treatments

The calves used in this experiment fit the definition of clinically healthy high-risk beef calves, described by Carrillo-Muro et al. [10]: (1) unknown health and management background; (2) weight at arrival between 150 and 200 kg; (3) age between 5 and 7 months; (4) weaned for a maximum of 14 d; (5) exposed to handling and transportation; (6) commingled with calves from different sources; and (7) unvaccinated upon arrival to feedlot. Continental × British crossbred bull calves (*n* = 461) were obtained from a dual-purpose, cow-calf grazing extensive system. All calves were sourced at an order-buying facility in Milpillas de la Sierra, Valparaiso, Zacatecas, located 120 km (~4 h) from the Torunos Livestock Preconditioning Center. Calves were transported in Kenworth cattle hauling model 2014 vehicles with a minimal space of 2 m^2^/calf. The total calves were received in three different periods; therefore, each arrival date represented each block in the experimental model as follows: 1 June 2023 (block 1, *n* = 137), 6 November 2023 (block 2, *n* = 166), and 11 April 2024 (block 3, *n* = 158).

**Figure 1:**
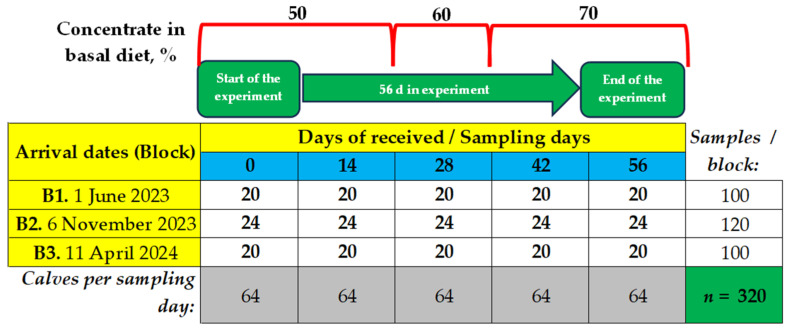
Received dates (3, block) and sampling schedule performed at 0, 14, 28, 42, and 56 d from reception to feedlot (five samplings).

A total of 64 of the 461 calves received qualified as “clinically healthy” by an experienced veterinarian (considering alertness, vigor, absence of dehydration, diarrhea, cough, and nasal or ocular discharge) and presented physiological parameters within normal ranges (cardiac auscultation > 86 and <125 beats per minute; normal lung auscultation > 20 and <44 breaths per minute; normal rectal temperature > 38 and <39.5 °C; normal mucosal color). The distribution of included calves per date was as follows: Block 1: 1 June 2023 (*n* = 20); Block 2: 6 November 2023 (*n* = 24); and Block 3: 11 April 2024 (*n* = 20). On average, the calves weighed 148.3 ± 1.3 kg BW, were 5.5 ± 0.5 months old, and had a rectal temperature of 38.7 ± 0.3 °C. Calves received on each date were placed (20 calves in June and March, or 24 calves in November) in group soil-surfaced outdoor pens (30 × 20 m) with a minimal space of 15 m^2^/calve, 0.45 cm of feed bunk space/calve, and 4 m^2^ shade/calve and with a shared drinker with float (2.20 × 0.80 cm, 1700 L cap), for this purpose, ensuring that each blood sampling period (0, 14, 28, 42, and 56 d) was replicated three times (once each date). The experimental design employed was a generalized complete block design, as shown in Figure 1.

### 2.2. Blood Sampling

Generally, the period of reception is established in 56 d (8 weeks). The sampling time points were determined by convenience by dividing every 14 d, resulting in 5 sampling times. This sampling criterion has been extensively applied in studies on newly received cattle.

Blood samples (*n* = 320) were collected from the jugular vein. Two blood tubes were collected from each calf: one 5-mL tube without a blood clotting agent (BD Vacutainer^®^, Fisher Scientific Mexico S. de R.L. de C.V., Mexico City) and another 5-mL tube with an anticoagulant (BD Vacutainer SST^®^, with EDTA K2-Dikysa, Fisher Scientific Mexico S. de R.L. de C.V., Mexico City). The clotted blood samples were centrifuged at 2500× *g* for 15 min at 4 °C. After collection, enzymatic activity, metabolites, and hematological response samples were stored on ice and transported directly to the university laboratory approximately 40 km from the experimental facilities. Upon arrival, the samples were immediately analyzed (within 2 h of collection).

Enzymatic activity and metabolites were quantified using an automated analyzer (Fuji Dri-Chem NX500^®^; Fujifilm, Tokyo, Japan). The following parameters were determined: (1) enzymatic activity: alkaline phosphatase (ALP), gamma glutamyltransferase (GGT), aspartate aminotransferase (AST), and alanine aminotransferase (ALT); (2) levels of total protein (TP), albumin (ALB), globulin (GLO = TP − ALB), ALB/GLO ratio, blood urea nitrogen (BUN), and creatinine (CRE); (3) total bilirubin (TBIL), total cholesterol (TCHO), and triglycerides (TG); (4) calcium (Ca) and glucose (GLU); and (5) electrolytes: sodium (Na^+^), potassium (K^+^), and chlorine (Cl^−^).

Whole blood samples were analyzed for hematological responses using an automatic cell counting machine (Exigo Veterinary Haematology Analyser^®^, Boule Medical AB, Spånga, Sweden). The following parameters were determined: (1) total white blood cells (WBC): lymphocytes (LYM), lymphocytes % (LYM%), monocytes (MON), monocytes % (MON%), granulocytes (GRA), and granulocytes % (GRA%); (2) platelets (PLT) and mean platelet volume (MPV); (3) red blood cells (RBC): red blood cell distribution width test % (RDW%), hematocrit (HCT), and mean corpuscular volume (MCV); and (4) hemoglobin (HGB): mean corpuscular hemoglobin (MCH) and mean corpuscular hemoglobin concentration (MCHC).

### 2.3. Statistical Analysis

All statistical analyses were performed using the page web “SAS OnDemand for Academics” (SAS Institute Inc., SAS Campus Drive, Cary, NC, USA). All parameters were checked for normal distribution using the procedure “PROC UNIVARIATE”. The data were tested for normality using the Shapiro–Wilk test. This study was a generalized complete block design with three arrival date blocks representing different received dates (block 1: June 2023; block 2: November 2023; and block 3: April 2024). The PROC MIXED procedure of SAS was used, involving a model that took into account the effects of days in reception (days of received, 0 14, 28, 42, and 56). One-way ANOVA was performed, and Bartlett’s test was used to assess variance homogeneity. There were 64 calves per sampling day (5 samplings = 320 total samples) used to quantify blood variables. The calf was the experimental unit for all dependent variables. When significant effects were detected, mean comparisons were conducted using the Tukey method with the LSMEANS instruction. Orthogonal polynomials were applied to evaluate linear and quadratic responses on different days of receipt. The effects of the number of days after being received were considered statistically significant at *p* ≤ 0.05.

## 3. Results and Discussion

The aim of this study was to determine the effect of days from arrival (0, 14, 28, 42, and 56) on enzymatic activity, metabolites, and hematological responses in a clinically healthy population of high-risk beef calves. Reference intervals (RIs) in hematology diagnostics are essential for defining a healthy animal in an experiment and for distinguishing and validating diagnoses in cattle practice and specialized bovine clinics. It is important to note that although all hematological parameter values measured in the current study were always within the established RIs by Carrillo-Muro et al. [10], according to the observed results, it appears that at least 42 d are necessary for calves to have the most adequate physiological and metabolic conditions before starting the fattening phase. On the other hand, as planned, all calves involved in the study fell in the “clinically healthy” category.

### 3.1. Enzymic Activity

The days of reception had no effect on the RIs of the enzymatic activity of ALP (14.4–469.6), GGT (10.0–47.9), AST (38.9–124.2), and ALT (16.6–44.4) [10] as shown in Table 1. ALP enzyme activity increased as the number of days from receiving increased (linear effect, *p* = 0.0001), significantly increasing from the 28th day (*p* ≤ 0.05). Since calves are growing, increases in serum ALP throughout the days could be mainly related to bone growth and osteoblast proliferation [11]; furthermore, total serum ALP activity is also composed of several soluble ALPs released by the membranes of different tissues such as the liver, kidney, and placenta [12,13,14]. Phosphatase activity in the blood serum of cattle tends to be low during hypomagnesemia; this condition can be highly prevalent when cattle come from magnesium-deficient regions [15]; thus, ALP can be used as an indicator of this deficiency. Our result is consistent with the ALP behavior reported for heavier newly received calves reared in Texas, USA, which were sampled every 14 d for 56 d from their arrival [7]. The activities of GGT and AST were greater on day 0 and on day 56 of receiving (quadratic effect, *p* = 0.001). The increases in GGT at the beginning and end of the reception period can be attributed to an increase in hepatic activity in these two stages [16]. While elevated AST levels at the beginning of reception may be associated with the catabolism of highly metabolic tissues (cardiac muscle, liver cells, and skeletal muscle cells) [7,17], they may also be due to nutrient deficiencies related to muscle, such as vitamin E or selenium (Se) [18]. The latter may be common in calves originating from pastures from soils deficient in Se or in the presence of Se antagonists in feeds, which can result in a low intake of Se that promotes low Se blood levels in cattle. In the present report, there was no history of Se deficiency in the region from which the calves in our study came. Smock et al. [7] reported similar enzymatic activity of AST to our results when a group of newly received calves was evaluated during a study that lasted 42 d. On the other hand, the increase in AST activity at 56 d may be due to improvements in feed efficiency of calves at the end of the reception stage [19]. Finally, ALT enzyme activity increased with the number of days of reception (linear effect, *p* = 0.0001), rising significantly from day 42 of reception (*p* ≤ 0.05). Increases in ALT levels may be a result of tissue damage, low perfusion in muscle tissue, reduced heat dissipation, hypoxia, and fatigue [20]. Therefore, ALT concentration can be used as an indicator of the well-being (or suffering) of calves during transportation. In general, the increase in liver enzyme activity is due to the increase in liver activity in view of nutritional changes, that is greater nutritional intake, increased growth rates, and feed efficiency [16,19].

### 3.2. Metabolites

**Figure 2:**
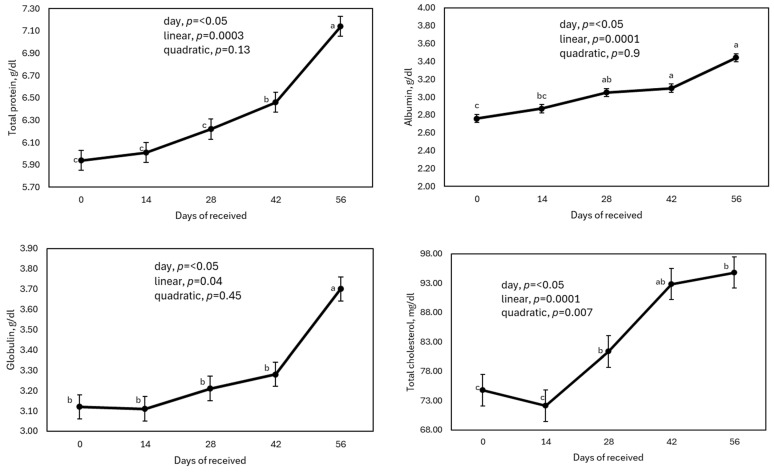

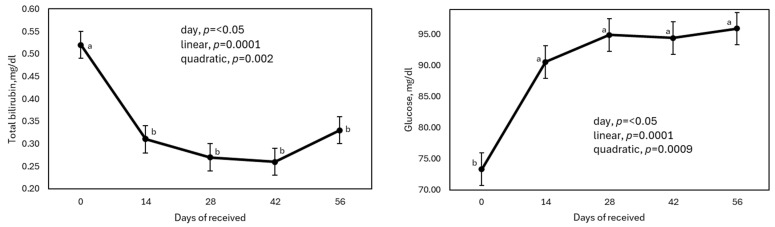
Effect of days after being received (0, 14, 28, 42, and 56 d) on metabolites of high-risk beef calves “clinically healthy”. ^a–c^ Means a row with different superscripts differ (*p* ≤ 0.05) according to Tukey’s test.

The metabolite concentration behavior during the first 56 d after arrival is shown in Table 2 and Figure 2. Except for BUN (without changes), CRE, and TBIL (which were linearly decreased), the rest of the metabolites measured were linearly (*p* ≤ 0.04) increased as the days passed since arrival. It is relevant to note that most of the metabolites did not show significant differences between 42 and 56 d. All metabolites stayed within normal RIs of TP (4.4–7.71), ALB (1.9–3.7), GLO (2.2–4.11), ALB/GLO ratio (0.68–1.32), BUN (6.91–16.1), CRE (0.52–1.35), TBIL (0.20–1.30), TCHO (50.0–127.7), TG (10.00–33.00), Ca (7.12–12.5), and GLU (26.1–126.0) [10].

#### 3.2.1. Total Protein, Blood Urea Nitrogen and Creatinine

The lowest values for TP, ALB, and GLO were observed at arrival; these values linearly increased as well as days from reception increased, being maximal at 56 d (linear effect, *p* ≤ 0.04). It has been reported that cattle have lower concentrations of these metabolites upon arrival at the feedlot, which subsequently increased when evaluated 42 d after arrival. TP was related to serum ALB and GLO concentrations. Therefore, the low TP value at arrival could indicate a low concentration of immunoglobulins (GLO) when arriving at the feedlot, followed by a GLO value increase from vaccination on d 28 or from a natural infection during these periods [7]. The increase in ALB concentration over time can be explained by greater protein intake. Generally, dietary protein concentration in reception diets can reach 15–16% of the CP of medium-high quality protein sources. Thus, increases in TP could also be associated with adequate availability and digestibility of microbial proteins [21,22,23]. The ALB:GLO ratio was linearly increased from 0.89 to 0.96 through the time. It has been informed that reference values for ALB:GLO ratio in healthy cattle from 0.80 to <1.0 were reported [24]. Values below the proposed range may be caused by physiological imbalances, including inflammation, immunodeficiency, and liver and kidney diseases.

There was no effect of the number of days on serum BUN levels. The results regarding BUN concentration behavior in newly received calves have been inconsistent. For example, Smock et al. [7] reported that newly received calves showed greater BUN concentrations at reception in the feedlot when compared to BUN concentrations measured after 42 d of receiving. On the contrary, Richeson et al. [6] observed the lowest values upon arrival (0 d) of calves, but after day 14, they showed higher BUN values. The inconsistencies observed in plasmatic BUN concentrations during the first weeks in the feedlot could be related to protein catabolism during the first days of arrival at the feedlot, which could indicate that calves release protein reserves to compensate for poor CP intake or inadequate CP content of the diet [6,25]. However, low BUN values after weeks of arrival could be explained by inadequate CP content in the receiving diet [26].

In concordance with that reported by Smock et al. [7], the serum CRE concentration was linearly reduced (*p* = 0.0001) as the number of days of receiving increased. CRE concentrations are a physiological indicator of the renal glomerular filtration rate, and high serum CRE levels beyond the normal range are indicative of nephritis [27]. On the other hand, lower CRE concentrations under the normal range indicate prolonged active catabolism of tissue proteins [28], such as in emaciated cattle, those with little muscle mass [11,27,29], or in animals subjected to excessive exercise (i.e., walking long distances to obtain pasture when they are grazing) [21].

#### 3.2.2. Total Bilirubin, Total Cholesterol and Triglycerides

Calves showed greater values of TBIL upon arrival at the feedlot; this value was linearly reduced (linear effect, *p* = 0.0001) as the days at the feedlot passed. TBIL is commonly used as a biomarker of liver status in cattle [30,31]. High levels are related to a negative energy balance, which is observed after long-distance transport and prolonged fasting period [32,33]. Therefore, high levels of TBIL in newly arrived calves are expected. A high level of TBIL upon receipt of cattle, with a gradual reduction of TBIL in the first 42 d in the feedlot, has been reported by Smock et al. [7]. A low TBIL level indicates a healthy liver [34,35]. The concentration of TCHO increased with the number of days of reception (linear effect, *p* ≤ 0.04), reaching its maximum at 56 d. Although TG concentration did not show a linear or quadratic tendency (*p* ≥ 0.21) when analyzed by means values, calves on day 56 had a greater TG concentration (*p* < 0.05) compared to the rest of the tested days. The observed increase in the serum concentrations of TCHO and TG at the end of the reception process may be an indication that the calves covered their energy requirements with the type of diet consumed at this stage [36,37].

#### 3.2.3. Calcium

Serum Ca linearly increased from 9.68 mg/dL at day 0 up to 11.63 mg/dL at day 56. Increases in serum Ca could be due to the fact that calves receiving diets were formulated to reach or surpass Ca requirement [10]; in the same manner receiving diets containing adequate quantities of vitamin D, both conditions promote an increase in mineral Ca in blood [38]. The same response to increased Ca plasma concentration was reported by Smock et al. [7] when evaluating Ca concentration in calves on day 0 vs. day 42 of receiving.

#### 3.2.4. Glucose

A dramatic increase in GLU was observed from day 14 of receiving, and this concentration was maintained until day 56. Similarly, Smock et al. [7] observed a significant GLU increase, but they registered an increase in GLU from day 28 of receiving. Lower GLU concentration upon arrival to a feedlot is mainly explained by the period of fasting that cattle are subjected to during their move to fattening facilities [39]. The increase in blood GLU suggests an improvement in energy balance associated with an improvement in energy intake during the receiving period [40].

#### 3.2.5. Electrolytes

The effect of the number of days after being received (0, 14, 28, 42, and 56 d) on electrolyte (Na^+^, K^+^, and Cl^−^) serum concentration is shown in Table 3. All electrolytes in all days measured were within RIs for Na^+^ (98.2–143.0), K^+^ (3.11–8.59), and Cl^−^ (71.1–109.0) [10], but the concentration showed a slight reduction as days of receiving passed (linear effect, *p* = 0.02). The effect of days on the reduction in electrolyte concentration was evident from day 14, and from day 28 onwards, there were no significant changes. Our results are contrary to those reported by Smock et al. [7], who noted that Na^+^ and K^+^ concentrations increased, being maximal at day 56 of receiving. Considering that plasma electrolyte concentrations were maintained throughout the evaluation period within the RIs, the slight decrease in electrolyte concentration as days passed, observed in the current study, is more of academic than practical importance. Even so, these slight variations can be explained, in part, by a condition of slight dehydration and stress upon the arrival of the calves (since these calves were weaned and transported at the time). This condition initially leads to higher concentrations of Na^+^, Cl^−,^ and K^+,^ which diminish as the calf rehydrates and adapts to the new environment [41].

### 3.3. Hematological Responses

**Figure 3:**
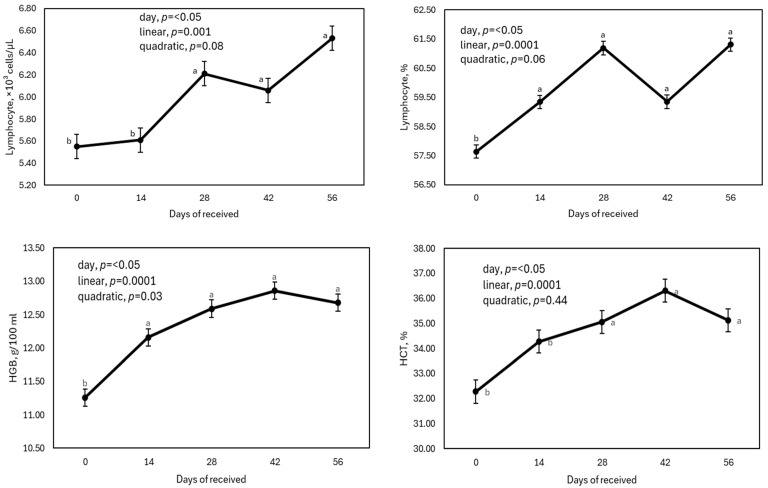

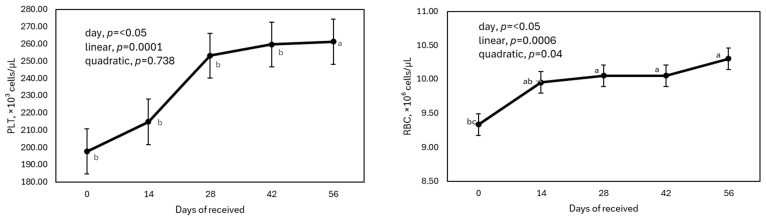
Effect of days after being received (0, 14, 28, 42, and 56 d) on blood cells of high-risk beef calves “clinicaly healthy” ^a–c^ Means a row with different superscripts differ (*p* ≤ 0.05) according to Tukey’s test.

The effect of days after being received (0, 14, 28, 42, and 56 d) on blood cells and platelets is shown in Table 4 and Table 5 and Figure 3. All blood cells in all days measured were within RIs [10]: (1) WBC (4.6–15.2), LYM (2.6–9.0), LYM% (33.6–74.61), MON (0.30–1.40), MON% (5.54–12.0), GRA (1.10–6.74), and GRA% (17.51–85.57); (2) PLT (91.2–444.9), and MPV (6.14–9.08); (3) RBC (7.88–11.90), RDW% (19.11–30.13), HCT (26.65–41.93), and MCV (29.1–41.8); (4) HGB (9.40–14.4), MCH (10.6–14.4), and MCHC (30.58–38.93).

#### 3.3.1. White Blood Cells

Counts of WBC, GRA, and MON were not affected by reception days (*p* > 0.05) and were within the established RIs [10]. WBC counts during receiving and through the first days in the feedlot in newly arrived calves are highly variable and can be affected by several factors, including disease, environment, and vaccination. In the specific case of newly arrived calves, increases in WBC count can be interpreted as a response to stressful conditions or greater immune adaptation to the environmental antigen challenge. For example, Smock et al. [7,9] performed consecutive studies (lasting 42 or 56 d) in newly arrived calves; they observed quadratic tendencies being maximal at day 0 and on the final day of the study (42 or 56). This response was similar when calves received or did not receive metaphylactic treatment. The calves included in Smock et al.’s [7,9] studies had heavier weights at arrival (220 and 258 kg BW) than those used in the current study (148 kg BW); in addition, they were transported a greater distance before arrival. Relying on findings by Richeson et al. [42], those researchers attributed WBC changes to the potential chronically elevated glucocorticoid levels as a result of stressful conditions prevailing during their studies. Other studies have reported increases in WBC counts only at the end of the receiving period [6,8,20]. These higher total WBC counts may also indicate that an animal has a greater capacity to mount an innate or adaptive immune response to a foreign antigen (improved immune status), which is a key goal of vaccination [8,43].

The LYM counts (expressed as cell/mL or as percentage) slightly increased (5 and 15%, respectively) with the number of days of reception (linear effect, *p* ≤ 0.001; Figure 3). Increases in LYM counts 4 to 8 weeks after the calves were received are a consistent response [6,7,8,9,33,43,44]. However, in some instances, no changes in LYM counts were detected [45]. In high-risk beef calves, low LYM counts may indicate viral or bacterial infection, chronic renal failure, or a common response after the use of corticosteroids [46]. Calves with acute stress (and higher glucocorticoid levels) promote LYM destruction and have a lower count of this parameter [47].

Throughout the time passed after arrival, the concentration of MON decreased when expressed as a percentage but not when expressed as cell/mL. This is in concordance with the findings of Smock et al. [7,9], who reported that the highest values for MON were on the last days of receiving (42 and 56 d). On the other hand, Richeson et al. [6] observed the highest values for MON both at the beginning and end of the receiving period (42 d). An increase in the number of MON has been reported during acute stress situations, such as after transport [33,48,49] or during the recovery phase of acute and chronic infections [50,51].

#### 3.3.2. Platelets

Both PLT and MPV values, when compared to day 0, were greater when measured on the final day from arrival. Platelets play an essential role in hemostasis. In practice, a count of platelets might be indicated with severe hemorrhage or increased bleeding tendency. An increased platelet count might be associated with an increased risk of thrombosis [46]. Thus, PLT counts are indicative of thrombosis or thrombocytopenia. Both conditions can be caused by parasites (i.e., anaplasmosis and leptospirosis), viruses (BVDV infection), and certain toxins (mycotoxins). On the other hand, recently, it has been reported that MPV may be a useful biomarker in calves with sepsis [52]. Our result is in agreement with that of Smock et al. [7]. However, other studies have indicated that PLT and MPV are higher during the first days of receiving [6,7,8,44]. We do not have an explanation for these inconsistencies, but as mentioned above, these changes were always maintained within the normal RIs.

#### 3.3.3. Red Blood Cells

There was a slight increase (linear effect, *p* = 0.0006) in RBC and MCV as the number of days of receiving increased; therefore, RDW% also increased. Lower values, but within the normal range, of RBC on day 0 of receiving have been observed previously, but similar to our results, this value increased as the days of receiving increased [7]. However, the opposite trend has also been observed, since newly arrived calves showed greater values of RBC on day 0 compared to RBC values on day 42 or day 56 [6,7,8,44]. These slight changes (up or down) in RBC values can be attributed, among other causes, to the status of hydration at the moment the sample was taken [53], but values under the normal range can indicate a predisposition to bovine respiratory disease [54]. Meanwhile, RDW% is associated with increased inflammation and decreased levels of antioxidants (low values) or vitamin and Fe deficiency (high values) [55]. Likewise, Smock et al. [7] reported an increasing trend in MCV as the number of days passed since receipt. MCV provides information about the volume or size of individual RBCs [56], and values outside the normal range can be related to certain minerals and vitamin deficiencies.

HGB concentration increased from 11.26 g/100 mL at arrival to 12.86 g/100 mL on day 56 of cattle stay (linear effect, *p* = 0.0001). Higher concentrations of HGB on the final days of reception (42 to 56 d) have been reported in several studies [6,7,8,9,44]. However, this trend is not always the same. For example, similar to our results, Smock et al. [7] observed a linear trend of increase, whereas other studies indicated a quadratic trend, with the highest concentration values, both registered on day 0 and the final day of the study (day 42 or 56) [6,8,9,44]. On the contrary, Avila-Jaime et al. [32] observed the highest concentration values during the first 2 weeks of receiving. HGB values below the normal range measured at the reception of high-risk newly received calves indicate anemia, but this can be resolved in the first 14 d [57]. It is possible that despite the fact that one of the most common causes of anemia may be iron deficiency [17,58], a lower HGB can also indicate a lack of amino acids, vitamins (especially vitamins B_12_, E, folic acid, and niacin), and/or minerals, which can be resolved by the processing of cattle at arrival or by supplementation strategies at the reception of cattle [59].

Values of MCH and HCT% increased linearly (*p* = 0.0001) with the days of the calves’ stay, but MCHC values did not change. Increases in MCH and HCT% without changes in MCHC values have been previously observed when newly arrived calves were sampled on day 0 and later on day 42 [9]. However, other studies reported that the MCHC value was highest at the beginning and at the end of receipt [7] or only upon arrival [6,8,32,43,44]. Generally, low HCT% values for RIs in high-risk newly received calves are related to dehydration [17], which is a common condition in this type of animal [46,60]. To reverse this dehydration, free access to water is necessary for more than 24 h after arrival [61]. However, iron deficiencies can cause lower values of HCT as well. The HCT values observed here on day 0 were within the normal range; the slight increases observed over time could be related to dietary effects [62].

It is important to highlight that this study was performed using specific characteristics of the calves that arrived as well as specific environmental conditions. Factors such as calf management prior to transportation, prevalence of certain illnesses in the place, time of transportation, feedlot facilities, type of cattle processing at arrival, and quality of receiving diet could affect the behavior of blood parameters in different ways. Thus, although the findings obtained here are valuable, they should be carefully extrapolated in a general way.

## 4. Conclusions

To our knowledge, this study is the first to establish the effects of reception days on the physiological, nutritional, metabolic, and clinical status of clinically healthy, high-risk beef calves during their first days of arrival at the feedlot. However, all parameters were within the RIs during the monitoring period. As the number of days after arrival increased, serum parameters related to health and immunity increased and metabolites related to tissue injury decreased. In contrast, the plasma electrolytes did not change. Apparently, under the conditions in which the study was carried out, at least 42 d is the minimum period after arrival to allow calves to reach the most adequate physiological and metabolic conditions before starting the fattening phase.

## Figures and Tables

**Table 1:** Effect of days after being received (0, 14, 28, 42, and 56 d) on enzyme activity of high-risk beef calves “clinically healthy”.

Item ^A^	Days After Being Received					Reference Intervals ^B^	SEM ^C^	Effects (*p*-Value)	
	0	14	28	42	56			Linear	Quadratic
ALP, U/I	175.66 ^b^	173.24 ^b^	240.95 ^a^	236.84 ^a^	234.68 ^a^	14.4–469.6 (209.7 ± 107)	12.52	0.0001	0.9400
GGT, U/I	19.86 ^a^	14.86 ^b^	15.30 ^b^	17.21 ^b^	19.27 ^a^	10.0–47.9 (17.0 ± 8.9)	1.06	0.1100	0.0010
AST, U/I	77.73 ^a^	55.71 ^c^	66.98 ^b^	72.73 ^b^	81.67 ^a^	38.9–124.2 (68.8 ± 21.9)	2.47	0.7300	0.0001
ALT, U/I	25.48 ^c^	22.34 ^c^	27.24 ^b^	30.49 ^a^	32.16 ^a^	16.6–44.4 (26.5 ± 7.3)	0.76	0.0001	0.0100

^A^ ALP = alkaline phosphatase, GGT = gamma glutamyltransferase, AST = aspartate aminotransfer-ase, ALT = alanine aminotransferase. ^B^ Reference intervals reported here are from the publication by Carrillo-Muro et al. [10]. ^C^ SEM = standard error of the mean. ^a–c^ Means a row with different superscripts differ (*p* ≤ 0.05) according to Tukey’s test.

**Table 2:** Effect of days after being received (0, 14, 28, 42, and 56 d) on metabolites of high-risk beef calves “clinically healthy”.

Item	Days After Being Received					Reference Intervals ^A^	SEM ^B^	Effects (*p*-Value)	
	0	14	28	42	56			Linear	Quadratic
Total protein, g/dL	5.94 ^c^	6.01 ^c^	6.22 ^c^	6.46 ^b^	7.14 ^a^	4.4–7.71 (6.22 ± 0.83)	0.09	0.0003	0.1300
Albumin, g/dL	2.76 ^c^	2.87 ^bc^	3.05 ^ab^	3.10 ^a^	3.44 ^a^	1.9–3.7 (2.97 ± 0.50)	0.06	0.0001	0.9000
Globulins, g/dL	3.12 ^b^	3.11 ^b^	3.21 ^b^	3.28 ^b^	3.70 ^a^	2.2–4.11 (3.18 ± 0.50)	0.06	0.0400	0.4500
Albumin/Globulins ratio	0.89 ^b^	0.93 ^b^	0.97 ^a^	0.96 ^a^	0.96 ^a^	0.68–1.32 (0.94 ± 0.17)	0.02	0.0050	0.3400
Blood urea nitrogen, mg/dL	11.40	11.23	10.90	11.11	11.77	6.91–16.1 (11.08 ± 2.31)	0.29	0.3600	0.5200
Creatinine, mg/dL	0.99 ^a^	0.78 ^b^	0.72 ^b^	0.72 ^b^	0.83 ^b^	0.52–1.35 (0.81 ± 0.20)	0.02	0.0001	0.0100
Total bilirubin, mg/dL	0.52 ^a^	0.31 ^b^	0.27 ^b^	0.26 ^b^	0.33 ^b^	0.20–1.30 (0.34 ± 0.29)	0.03	0.0001	0.0020
Total cholesterol, mg/dL	74.77 ^c^	72.12 ^c^	81.36 ^b^	92.85 ^ab^	94.80 ^a^	50.0–127.7 (78.6 ± 22.0)	2.66	0.0001	0.0070
Triglycerides, mg/dL	17.54 ^b^	22.25 ^b^	24.46 ^b^	25.30 ^b^	29.10 ^a^	10.00–33.00 (17.00 ± 6.90)	3.13	0.2100	0.3700
Calcium, mg/dL	9.68 ^c^	10.20 ^bc^	10.41 ^b^	10.65 ^b^	11.63 ^a^	7.12–12.5 (10.28 ± 1.42)	0.15	0.0001	0.3600
Glucose, mg/dL	73.32 ^b^	90.54 ^a^	94.41 ^a^	94.90 ^a^	95.90 ^a^	26.1–126.0 (89.0 ± 22.5)	2.61	0.0001	0.0009

^A^ Reference intervals reported here are from the publication by Carrillo-Muro et al. [10]. ^B^ SEM = standard error of the mean. ^a–c^ Means a row with different superscripts differ (*p* ≤ 0.05) according to Tukey’s test.

**Table 3:** Effect of days after being received (0, 14, 28, 42, and 56 d) on electrolytes of high-risk beef calves “clinically healthy”.

Item	Days After Being Received					Reference Intervals ^A^	SEM ^B^	Effects (*p*-Value)	
	0	14	28	42	56			Linear	Quadratic
Sodium, mEq/L	130.67 ^a^	125.45 ^b^	122.81 ^b^	126.54 ^b^	128.91 ^b^	98.2–143.0 (126.3 ± 12.1)	1.11	0.0200	0.0001
Potassium, mEq/L	5.85 ^a^	4.95 ^b^	4.61 ^c^	4.63 ^c^	4.37 ^c^	3.11–8.59 (4.93 ± 1.21)	0.12	0.0001	0.0600
Chlorine, mEq/L	95.64 ^a^	89.47 ^b^	87.71 ^b^	90.64 ^b^	90.45 ^b^	71.1–109.0 (90.8 ± 9.8)	0.93	0.0001	0.0001

^A^ Reference intervals reported here are from the publication by Carrillo-Muro et al. [10]. ^B^ SEM = standard error of the mean. ^a–c^ Means a row with different superscripts differ (*p* ≤ 0.05) according to Tukey’s test.

**Table 4:** Effect of days after being received (0, 14, 28, 42, and 56 d) on white blood cells of high-risk beef calves “clinically healthy”.

Item	Days After Being Received					Reference Intervals ^A^	SEM ^B^	Effects (*p*-Value)	
	0	14	28	42	56			Linear	Quadratic
Total white blood cells, ×10^3^ cells/μL	9.67	9.51	10.21	10.10	10.08	4.6–15.2 (9.65 ± 2.62)	0.30	0.1920	0.9700
Lymphocytes, ×10^3^ cells/μL	5.55 ^b^	5.61 ^b^	6.21 ^a^	6.06 ^a^	6.53 ^a^	2.6–9.0 (5.87 ± 1.6)	0.11	0.0010	0.0800
Lymphocytes, %	57.64 ^b^	59.34 ^a^	61.19 ^a^	59.35 ^a^	61.31 ^a^	33.6–74.61 (59.2 ± 9.48)	0.23	0.0001	0.0600
Monocytes, ×10^3^ cells/μL	0.89 ^a^	0.85 ^b^	0.84 ^b^	0.85 ^b^	0.87 ^b^	0.30–1.40 (0.83 ± 0.26)	0.03	0.4800	0.5020
Monocytes, %	9.00 ^a^	8.37 ^b^	7.88 ^b^	7.92 ^b^	8.01 ^b^	5.54–12.0 (8.28 ± 1.5)	0.18	0.0001	0.0700
Granulocytes, ×10^3^ cells/μL	3.21	3.03	3.18	3.24	3.38	1.10–6.74 (3.33 ± 1.42)	0.20	0.7600	0.5130
Granulocytes, %	33.34	32.34	31.05	32.65	32.12	17.51–85.57 (35.3 ± 14.8)	1.33	0.5200	0.2400

^A^ Reference intervals reported here are from the publication by Carrillo-Muro et al. [10]. ^B^ SEM = standard error of the mean. ^a,b^ Means a row with different superscripts differ (*p* ≤ 0.05) according to Tukey’s test.

**Table 5:** Effect of days after being received (0, 14, 28, 42, and 56 d) on platelets and red blood cells of high-risk beef calves “clinically healthy”.

Item ^A^	Days After Being Received					Reference Intervals ^B^	SEM ^C^	Effects (*p*-Value)	
	0	14	28	42	56			Linear	Quadratic
Platelets, ×10^3^ cells/μL	197.62 ^b^	214.73 ^b^	235.06 ^b^	259.55 ^a^	261.17 ^a^	91.2–444.9 (239.8 ± 90.7)	13.10	0.0001	0.7380
MPV, fL	7.031 ^b^	7.17 ^b^	7.17 ^b^	7.19 ^b^	8.25 ^a^	6.14–9.08 (7.22 ± 0.97)	0.15	0.4170	0.6440
RBC, ×10^6^ cells/μL	9.33 ^bc^	9.95 ^ab^	10.3 ^a^	10.05 ^a^	10.05 ^a^	7.88–11.90 (9.77 ± 1.07)	0.16	0.0006	0.0400
RDW, %	26.45 ^a^	27.03 ^a^	27.55 ^a^	26.71 ^a^	20.43 ^b^	19.11–30.13 (26.4 ± 2.54)	0.23	0.2700	0.0030
Hemoglobin, g/100 mL	11.26 ^b^	12.16 ^a^	12.59 ^a^	12.68 ^a^	12.86 ^a^	9.40–14.4 (12.05 ± 1.31)	0.13	0.0001	0.0300
Hematocrit, %	32.28 ^b^	34.27 ^b^	35.06 ^a^	36.31 ^a^	35.13 ^a^	26.65–41.93 (34.27 ± 3.92)	0.46	0.0001	0.4400
MCV, fL	34.31 ^b^	34.37 ^b^	35.33 ^b^	36.11 ^b^	39.41 ^a^	29.1–41.8 (35.12 ± 3.1)	0.35	0.0005	0.3480
MCH, pg	12.11 ^c^	12.24 ^b^	12.63 ^ab^	12.91 ^a^	12.82 ^a^	10.6–14.4 (12.36 ± 1.03)	0.14	0.0001	0.6420
MCHC, g/dL	35.34	35.64	36.04	35.66	35.84	30.58–38.93 (35.35 ± 1.98)	0.22	0.1100	0.0600

^A^ MPV = mean platelet volume, RBC = red blood cells, RDW% = red blood cell distribution width test %, MCV = mean corpuscular volume, MCH = mean corpuscular hemoglobin, MCHC = mean corpuscular hemoglobin concentration. ^B^ Reference intervals reported here are from the publication by Carrillo-Muro et al. [10]. ^C^ SEM = standard error of the mean. ^a–c^ Means a row with different superscripts differ (*p* ≤ 0.05) according to Tukey’s test.

## Data Availability

The information published in this study is available upon request from the corresponding author.
